# Adapting to the impact of the COVID-19 pandemic on a randomized controlled trial examining lifestyle behaviors on cognitive functioning in obese African American adults: Building Research in Diet and Cognition (BRIDGE)

**DOI:** 10.21203/rs.3.rs-290482/v1

**Published:** 2021-04-26

**Authors:** Marian Fitzgibbon, Lara Blumstein, Linda Schiffer, Mirjana A. Antonic, Andrew McLeod, Roxanne Dakers, Leo Restrepo, Elizabeth A. Boots, Jennifer C. Sanchez-Flack, Lisa Tussing-Humphreys, Melissa Lamar

**Affiliations:** a. Institute for Health Research and Policy, University of Illinois at Chicago, 1747 West Roosevelt Road, Chicago, IL 60608.; b. Department of Pediatrics, University of Illinois at Chicago, 1200 West Harrison Street, Chicago, IL 60607.; c. School of Public Health, University of Illinois at Chicago, 1603 W Taylor St, Chicago, IL 60612.; d. University of Illinois Cancer Center, University of Illinois at Chicago, 818 South Wolcott Avenue, Chicago, IL 60612.; e. Department of Kinesiology, University of Illinois at Chicago, 1919 W. Taylor St, Chicago, IL 60612.; f. Rush Alzheimer’s Disease Center, Rush University, 1750 West Harrison Street, Chicago, IL 60612.

## Abstract

**Background.:**

The COVID-19 pandemic has significantly altered the research landscape for clinical trials, requiring thoughtful consideration regarding how to handle the risks and benefits of continuing them.

**Design.:**

This brief report describes the experience of adapting the Building Research in Diet and Cognition (BRIDGE) study, a randomized clinical trial examining the effects of the Mediterranean Diet, with and without weight loss, on cognitive functioning in 185 older obese African American adults during the COVID-19 pandemic.

**Measurement.:**

The University of Illinois at Chicago (UIC) developed an expedited amendment process for research shifting to remote data collection. For the Cohort 3, 14-month data collection period, we adapted our protocol to allow data collection via telephone and e-mail. We were unable to collect certain measures that required face-to face contact.

**Results.:**

For measures that could be collected remotely, 14-month retention was similar for Cohort 3 and earlier cohorts: data were collected for 86.9% of cohort 3 (remote) and 87.9% of cohorts 1 and 2 (face to face), p = .84.

**Conclusions.:**

In order to preserve the integrity of our clinical trial and ensure the safety of our participants and staff during the COVID-19 pandemic, we had to carefully and efficiently adapt our data collection procedures. The procedures put in place allowed us to collect our primary outcomes and the majority of our secondary outcomes and will enable us to examine the role of dietary intake, with and without weight loss, on cognitive functioning in a vulnerable and high-risk population.

## INTRODUCTION

The COVID-19 pandemic has dramatically changed how we work, socialize, and function on a day-to-day basis. At academic medical centers across the United States (US), the COVID-19 pandemic has altered many rigorously planned research protocols of randomized clinical trials (RCTs)^1, 2^. At the time of this writing, there are 364,274 registered studies listed on ClinicalTrials.gov^3^. This includes 156,181 drug or biologic, 66,769 surgical or device, and 93,750 behavioral trials^3^. Also, at the time of this writing, this worldwide pandemic has infected more than 25,456,670 and killed more than 427,626 people in the US^4^. It was apparent that to preserve our trial’s integrity, we needed to quickly adapt our data collection procedures given the final data collection period of our three-arm RCT was scheduled to begin in April-May of 2020, and we were unable to collect data in-person.

Briefly, the Building Research in Diet and Cognition (BRIDGE) trial is a three-arm RCT with 185 obese African American older adults (55 – 85 years of age) randomized to: 1) a Typical Diet Control; 2) a Mediterranean Diet lifestyle intervention without caloric restriction (MedDiet-A); or 3) a Mediterranean Diet with caloric restriction to promote weight loss (MedDiet-WL). The trial was designed to test whether MedDiet-WL would produce better results than a MedDiet-A on the primary outcome of cognitive functioning and secondary outcomes of body weight, body composition, dietary intake, lifestyle behaviors, and cardiometabolic-related biomarkers. Both MedDiet lifestyle interventions were 14-months long, including an 8-month, 27-session group intervention, followed by a 6-month minimal contact period. The design and baseline characteristics of the sample are described in detail elsewhere^5, 6^.

Before beginning our final data collection period for our third and final cohort, many national organizations such as the National Institutes of Health (NIH), the Centers for Disease Control and Prevention (CDC), and the Office of Research and Development (ORD), had indicated research visits to be nonessential^7, 8^. For trials funded through the NIH, it was advised that investigators work with their respective Institutional Review Boards (IRBs) to assess the appropriate measures in order to guarantee the protection of both research participants and study staff. At the BRIDGE study site, the University of Illinois at Chicago (UIC) IRB instituted a hold on human subject research, including in-person, face-to-face interaction that was not critical from a patient care perspective. The IRB instructions, however, did indicate that research that could be conducted remotely was potentially acceptable.

The purpose of this paper is to document our experience of adapting the BRIDGE research protocol during the COVID-19 pandemic to meet data collection requirements, retention expectations, and ultimately, to answer our research questions. The BRIDGE trial is registered at ClinicalTrials.gov (NCT03129048).

## METHODS

The BRIDGE Trial was conducted in three cohorts of approximately 60 persons each^5^. Participants provided data at baseline, immediately after the 8-month intervention, and at 14 months. Data collection assessed sociodemographic status, cognition, body weight, body composition, dietary intake, physical activity, cardiometabolic-related biomarkers, and health history. Prior to the pandemic onset, we were on course to begin our 14-month follow-up data collection for cohort 3 starting in April-May 2020.

The primary outcome of the study was change in cognitive functioning from baseline to post 8-month intervention. The cognitive assessment protocol was a 60-minute neuropsychological protocol consistent with that developed by the National Institute of Neurological Disorders and Stroke and the Stroke-Canadian Networks Neuropsychology working group^9^. The secondary outcomes were related to body weight measured using a digital scale (Tanita, Arlington Heights, IL), whole-body composition using the General Electric Lunar iDXA machine (GE Healthcare, US), and cardiometabolic risk markers, including cholesterol, insulin, and glucose measured by Quest Diagnostics (Wood Dale, IL) Dietary intake was assessed using the Harvard Food Frequency Questionnaire^10^. Participants were also asked to wear the Actigraph GT3X triaxial accelerometer^11^ for 7 days to measure physical activity objectively. Functional capacity and mobility capacity were measured with the six-minute walk test^5, 12^.

After the onset of the pandemic and associated restrictions to in-person, face-to-face human subject research interaction, we immediately began to plan how we could complete the RCT given the amount of time and effort staff and participants had contributed already as well as the scientific value of completing the study. Simultaneously, the UIC IRB developed an expedited amendment process specifically for changes to research caused by the COVID-19 shutdown. The first step in adapting to the COVID-19 research restrictions was to review and verify the options we had for remote data collection. At the outset of the pandemic, only about half of our cohort 3 participants reported having reliable online access to face-to-face remote technology (e.g., Zoom) (www.Zoom.us). Therefore, we decided to conduct telephone interviews because all of our participants had phone access. To determine the feasibility of collecting data via telephone, staff practiced and timed this data collection procedure. Based on these practice interviews, it was decided that our 14-month follow up data collection for cohort 3 could be completed over two sessions (one hour each) via telephone: one call would include lifestyle measures and dietary intake data collection, the second call would collect data using a modified cognitive assessment protocol.

Data collection was conducted while all faculty and staff were working remotely. Cell phones, hot spots, and laptops were provided to the data collection team, as needed. The study coordinator received permission to enter the offices during the shut-down to prepare all data collection material for distribution to the data collection team; once compiled, it was safely dropped off with no contact. Once our new amendment, protocol, and the addendum to informed consent forms were finalized, submitted, and approved by the IRB, data collection began.

Most cognitive assessment measures were validated for remote collection^13^ and able to be administered in the same fashion as had been done during in-person, face-to-face visits. One measure, the Trail Making Test, was adapted for oral administration via telephone as previously described and validated^14, 15^. Two of the original BRIDGE neuropsychological protocol measures, i.e., Digit Symbol Coding^16^ and the Stroop Color Word^17^ measures, were excluded because a key aspect of administration involves the exchange of test forms for participant usage and completion (i.e., reading and/or writing). During practice interviews, we developed a detailed protocol for telephone cognitive assessments.

Data collection involving specific equipment was modified wherever possible to ensure capture of these important outcomes. For example, we were able to remotely collect participant weight by mailing digital scales to each participant. Additionally, the study coordinator brought the necessary equipment for initializing, charging, and downloading accelerometers to the home of a staff member, who mailed accelerometers to cohort 3 participants and maintained contact with them via text messaging for the requisite 7 days of wear. The accelerometers were then returned to the staff member’s home by US Mail. Because we were unable to meet participants in person, we did not collect blood samples, assess whole-body composition using iDXA, measure blood pressure, or assess functional capacity via the six-minute walk test.

## RESULTS

As seen in Table 1, study participants were at increased risk for COVID-19 due to age and co-morbid medical conditions. Specifically, the mean age was 66.4 years, and 26.2% were ≥ 70 years. While all of the participants were obese (body mass index (BMI) ≥ 30 kg/m^2^), 37.7% had class II obesity (BMI = 35 to <40 kg/m^2^), and 27.9% had class III obesity (BMI ≥40 kg/m^2^). Most participants had been diagnosed with high blood pressure (68.9%), and many were diagnosed with high cholesterol (46.5%) and type 2 diabetes (23.0%).

As shown in Table 2, participation in remote data collection by Cohort 3 at 14 months (final data collection) did not significantly differ from participation in face-to-face, in-person data collection by Cohorts 1 and 2 at 14 months. The only exception was the collection of accelerometer data, though the difference was not statistically significant (63.9% for C3 vs. 77.4% for C1+C2, p=0.052). As noted above, we could not collect blood samples, assess body composition, blood pressure, or complete the six-minute walk test.

## DISCUSSION

COVID-19 will not be the last regional, national, or international disruption that affects day-today life^18^. Clinical research studies must have the ability to adapt to such disruptions while respecting safety and confidentiality of participants and staff. Researchers have an obligation to implement and measure the feasibility and scalability of new approaches and the impact of these new approaches on participants and communities. These data will provide the basis for further guidelines regarding how to conduct research during times of crisis^1^.

The health inequities in the US that impact minority communities existed prior to the COVID-19 pandemic^19^. In Chicago, African Americans make up 30% of the population; yet, they represent 50% of COVID-19 cases and approximately 70% of COVID-19 deaths, most of which are concentrated in the most vulnerable communities^20^. Given our involvement in these communities pre pandemic and the trusting relationships we had built between staff and BRIDGE participants, there was strong motivation to complete our research work together. Cohort 3 participants had already completed the full 8-month intervention and post-intervention data collection. We clearly communicated our appreciation for the time participants were taking to provide follow-up data while living with the pandemic’s ongoing day-to-day challenges. For example, we conducted wellness calls and discussed relevant non-research related issues such as COVID-19 safety precautions. In our interactions with our older adult participants, many of whom lived alone, most reported remaining at home and in contact with friends and family via phone and text messaging.

In retrospect, there could have been changes to data collection that may have been easier for participants. For example, many participants reported phone interview fatigue, as data collection lasted approximately two hours over two sessions. Perhaps participants could have self-administered their dietary intake and lifestyle measures via either a postage-paid return envelope system or an online portal. We could have further explored using online platforms, but not all participants had regular online access, and at the beginning of the pandemic, there were some concern regarding maintaining confidentiality. However, since the start of the pandemic, many of these video platforms have increasingly improved their security measures. Additionally, given that most of our participants had smartphones, we could have further explored using RedCap surveys online. Nevertheless, compared to in person assessments pre-pandemic that required multiple visits to our offices and a minimum of three hours of data collection, remote assessments were likely easier for older participants. This could portend the greater use of remote assessments even after the COVID-19 pandemic ends.

The COVID-19 pandemic will continue to disrupt research activities for months to come. Researchers need to utilize existing communication platforms (e.g., text messaging, email, and remote data collection) while, first and foremost, protecting the safety of study participants and staff^18^. We are currently analyzing our data, having successfully collected the majority of our primary outcome cognitive data and our secondary weight, dietary intake, physical activity, and lifestyle behavior data. Given the dearth of effective pharmacological treatments to prevent or halt cognitive decline, particularly in at-risk obese African American older adults^21–23^, and even int the face of this global pandemic, this study has the potential to provide important insights regarding the role of lifestyle in maintaining cognitive health in aging populations^24–26^.

## Figures and Tables

**Table 1.** Participant characteristics at baseline, Cohort 3

	N	Mean or %	SD or N
Age at randomization, yr	61	66.4	(6.2)
55–69		73.8%	(45)
≥70		26.2%	(16)
Gender	61		
Female		93.4%	(57)
Male		6.6%	(4)
Race	61		
Black or African-American, not Hispanic		91.8%	(56)
Hispanic		1.6%	(1)
Native American		1.6%	(1)
Multiracial		4.9%	(3)
Marital status	61		
Single		13.1%	(8)
Married		36.1%	(22)
Widowed		21.3%	(13)
Divorced		29.5%	(18)
Medical conditions			
High blood pressure	61	68.9%	(42)
High cholesterol	61	47.5%	(29)
Type 2 diabetes	61	23.0%	(14)
Sleep apnea	61	24.6%	(15)
Weight, kg	61	102.1	(15.3)
Height, cm	61	164.5	(6.8)
BMI, kg/m^2^	61	37.8	(5.5)
BMI category	61		
Obesity class I (30-<35 kg/m^2^)		34.4%	(21)
Obesity class II (35-<40 kg/m^2^)		37.7%	(44)
Obesity class III (≥40 kg/m^2^)		27.9%	(17)
Percent body fat	60	48.5	(5.1)

**Table 2.** Participation in 14-month^a^ Data Collection by Cohort 3 Compared to Cohorts 1 and 2

	C3(N=61)		C1+2(N=124)		
	%	N	%	N	p^b^
Any data^c^	86.9%	53	87.9%	109	0.84
Cognitive measures	77.0%	47	78.2%	97	0.86
Diet intake	86.9%	53	87.1%	108	0.97
Weight	82.0%	50	85.5%	106	0.54
Lifestyle questionnaires	86.9%	53	87.9%	109	0.84
Accelerometer	63.9%	39	77.4%	96	0.052

a Data collection for the 14-month visit ran from 6/2020–9/2020 for Cohort 3, from 5/2019–9/2019 for Cohort 2, and from 6/2018–10/2018 for Cohort 1.

b From chi-square tests for differences between Cohort 3 and Cohort 1+2.

c Includes only measures collected at the 14-month visit for Cohort 3: cognitive, diet, weight, questionnaires, accelerometer.
